# Opening the “Black Box”: Functions of the Frontal Lobes and Their Implications for Sociology

**DOI:** 10.3389/fsoc.2019.00003

**Published:** 2019-02-14

**Authors:** Rengin B. Firat

**Affiliations:** Department of Sociology, University of California, Riverside, Riverside, CA, United States

**Keywords:** frontal lobes, neurosociology, biosociology, prefrontal cortex, frontal lobe functions, social neuroscience, cognition

## Abstract

Previous research has provided theoretical frameworks for building inter-disciplinary bridges between sociology and the neurosciences; yet, more anatomically, or functionally focused perspectives offering detailed information to sociologists are largely missing from the literature. This manuscript addresses this gap by offering a comprehensive review of the functions of the frontal lobes, arguably the most important brain region involved in various “human” skills ranging from abstract thinking to language. The paper proposes that the functions of the frontal lobe sub-regions can be divided into three inter-related hierarchical systems with varying degrees of causal proximity in regulating human behavior and social connectedness: (a) the most proximate, voluntary, controlled behavior—including motor functions underlying action-perception and mirror neurons, (b) more abstract motivation and emotional regulation—such as Theory of Mind and empathy, and (c) the higher-order executive functioning—e.g., inhibition of racial bias. The paper offers insights from the social neuroscience literature on phenomena that lie at the core of social theory and research including moral cognition and behavior, and empathy and inter-group attitudes and provides future research questions for interdisciplinary research.

## Introduction

The notion that the mind, self, and society are fundamentally intertwined (Mead, 1934) is not new to sociology. Since the first acquaintance with the term “neurosociology” (TenHouten, 1973), biology, and neurosciences have slowly been finding their ways into sociology (e.g., Franks, 2010; Turner and Maryanski, 2012; Franks and Turner, 2013; Kalkhoff et al., 2016a; Melamed et al., 2017); albeit, facing much resistance, and opposition from the discipline (Hopcroft, 2016). On the one hand, this resistance is partly due to the biophobia fueled by the racist and sexist legacy of the Social Darwinist accounts of human biological functioning; on the other, it is in large part due to a lack of knowledge and information available to sociologists on how the human brain and biology operate. This lack of information, in conjunction with the growing importance of the field of neurosciences and especially the sub-field of social neurosciences, has led to concerns about neurosciences' oversight of the so-called “thicker,” sociological concepts and the philosophical roots of the concepts of study and their operationalizations (Abend, 2011, 2017) as well as the assumed causalities in the neuro-fields (from brain to behavior) and biomedicalization of culture and social domains like mental health, education, gender (Martin, 2004; Pickersgill, 2013). While previous literature has offered theoretical guidelines on bridging this gap between sociology and the neurosciences (e.g., Firat and McPherson, 2010; Firat and Hitlin, 2012; Kalkhoff et al., 2016b), more anatomically or functionally focused perspectives opening the so-called “black box” to the sociologists is largely missing from the literature.

This paper attempts to bridge this gap by offering a comprehensive review of the functions of the frontal lobes, arguably the most important brain region involved in various “human” skills ranging from abstract thinking to language, and their implications for causality and epistemological mechanisms in social theory (see for example, Abend et al., 2013; Vaidyanathan et al., 2016 for sociological discussions of causality). The frontal lobes, the largest brain region constituting almost one third of the entire human brain (Blumenfeld, 2002), extend from the central sulcus and lateral fissure to the frontal pole (Waxman, 2010). Potentially through a recursive cortical evolution between brain, environment, and behavior, the evolutionary development of the frontal lobes played a key role in primates and especially humans above other mammalians to develop many cognitive skills, including working memory and language. The frontal lobes of the primates, especially humans (particularly the prefrontal cortex) develop later in an individual's life and are disproportionately bigger than the frontal cortices of other species (Fuster, 2002). The frontal lobes are also extensively connected with other cortical and subcortical regions (Fuster, 2002). Consistent with its larger size and numerous connections with other brain regions, this brain area is responsible for a diverse and often contradictory set of functions that make it one of the most complex brain regions to study (Blumenfeld, 2002). The complexity of the frontal lobe functioning is also evident from the patients with damage to this area (i.e., vmPFC) who may perform within the normal range in standardized tests of intellectual ability and language, while having major problems in emotional regulation, moral functioning or complex, real life social behavior (Anderson et al., 1991, 2006). The frontal lobe functions include (but are not limited to) abstract reasoning, creativity, and socially appropriate behavior, which are critical for humans to engage in ongoing social interactions as well as sophisticated decisions (Stuss and Benson, 1984; Blumenfeld, 2002; Waxman, 2010).

**Figure 1:**
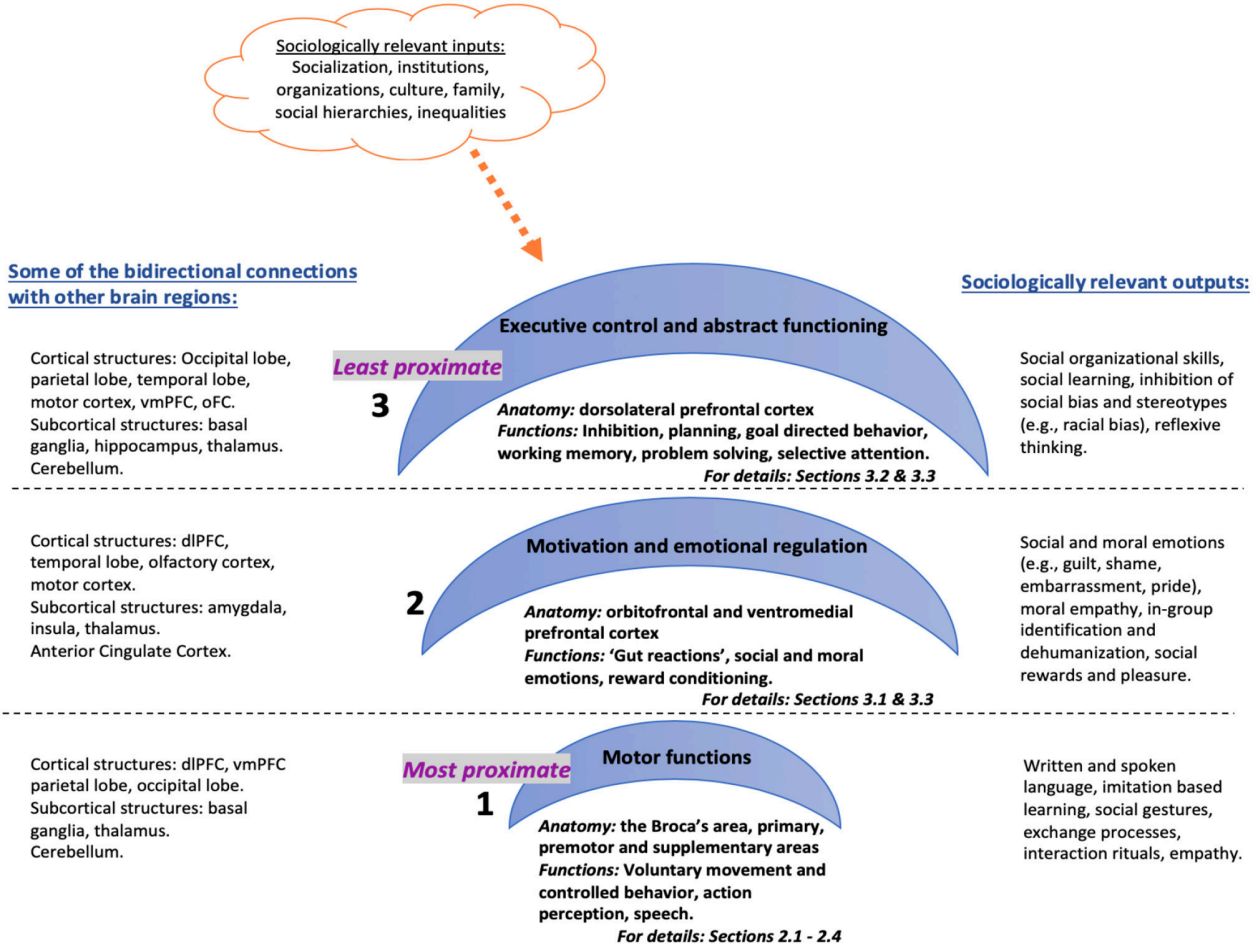
Three inter-related hierarchical systems with varying degrees of causal proximity in regulating human behavior and thought.

I propose that the anatomical architecture of the frontal lobes can be divided into three inter-related hierarchical systems with varying degrees of causal proximity in regulating human behavior and thought (see Figure 1). I argue that this anatomical structure implicates a functional hierarchy that has clear consequences for social behavior, particularly for social connectedness by regulating: (a) action-perception model of mimicry and basic empathy (aka mirror neurons), (b) emotional and cognitive empathy (aka Theory of Mind) and (c) self-reflexivity and overwriting automatic responses (e.g., inhibiting certain social responses or changing decisions based on reflexive evaluations). The first system consists of the most proximate operator areas, which are responsible for voluntary, controlled behavior that include motor functions such as speaking and muscle movements. Anatomical correlates are the Broca's area in the dominant sphere as well as the motor regions including primary motor, premotor and supplementary motor areas. One of the most remarkable (albeit controversial) discoveries within this region is the particular neurons firing when acting or observing the same act in another, thus speculatively facilitating action-perception or mimicry as precursors to empathy (Gallese, 2001; Iacoboni et al., 2005; Kilner et al., 2009). The second system involves the somewhat abstract, intermediary motivator regions involved in “gut reactions” and emotional regulation. Functions of this system would be reward processing, social or moral emotional regulation and empathy (Greene et al., 2001; Moll et al., 2001); anatomical correlates are the bottom half of the prefrontal cortex—i.e., the ventromedial prefrontal cortex. The third system is the least proximate, higher-order executive control center that reconciles person's behavior with the environment at an abstract level to connect past, present and future experiences (MacDonald et al., 2000; Alvarez and Emory, 2006). Functions of this system include as attention, working memory, planning, decision making, and the anatomical correlates are the top half of the prefrontal cortex, i.e., dorsolateral prefrontal cortex.

This anatomical and functional organization implies that the frontal lobes operate at three inter-dependent causal levels that range from immediate action, to conscious or non-conscious feelings, to mostly conscious higher-order evaluative but also inhibitory thoughts. Lieberman (2007) also suggests a similar division of labor between the ventromedial and lateral prefrontal cortices (among other brain regions), the former corresponding to an automatic social cognition system and the latter involved in a controlled social cognition system. The argument here is not that the brain causes social behavior or thoughts (i.e., ultimate causes), but rather that the brain is a proximate causal mechanism (the “how”) that catalyzes internal and external stimuli into individual action or thought (see Scott-Phillips et al., 2011 for the distinctions between proximate and distal causes in evolutionary theory). So, the paper does not seek to ascribe any agency to the brain, but rather proposes that it is potentially one of many proximate causal mechanisms—immediate, sufficient causes. For example, damage to the occipital lobes (the visual cortex at the back portion of the brain) would impair vision (a proximate cause), but damage to eyes would also impair vision (another proximate cause). But either one of these damages might have occurred due to a car accident the person has experienced (distal or ultimate cause).

Furthermore, human behavior and cognition result from neural network activity rather than isolated regional activations (Baronchelli et al., 2013). Frontal lobes are not the only brain areas related to some of the processes or functions discussed here (for example, temporoparietal junction, superior parietal lobe, and the pulvinar among others are all part of the attention networks of the brain, Raz, 2004). Yet, an understanding of how distributed networks operate require a foundational knowledge on the individual functions of anatomical regions. Therefore, in this paper, I offer a review of the functional organization of the frontal lobes as they are vital to higher cognitive and emotional operations that are required for complex human social organization. In the next sections, I demonstrate this organizational hierarchy by first explaining the contribution of the frontal lobes to voluntary, controlled behavior (motor functioning) and the mirror neuron system, and then elaborating on how the prefrontal cortex is involved in more abstract aspects of individual functioning including motivation and emotional regulation, and executive functioning, respectively. Lastly, I illustrate the implications of this organization for social theory by focusing on the research on: (a) moral cognition and behavior and (b) empathy and inter-group attitudes.

## Frontal Lobes and Voluntary, Controlled Behavior

**Figure 2:**
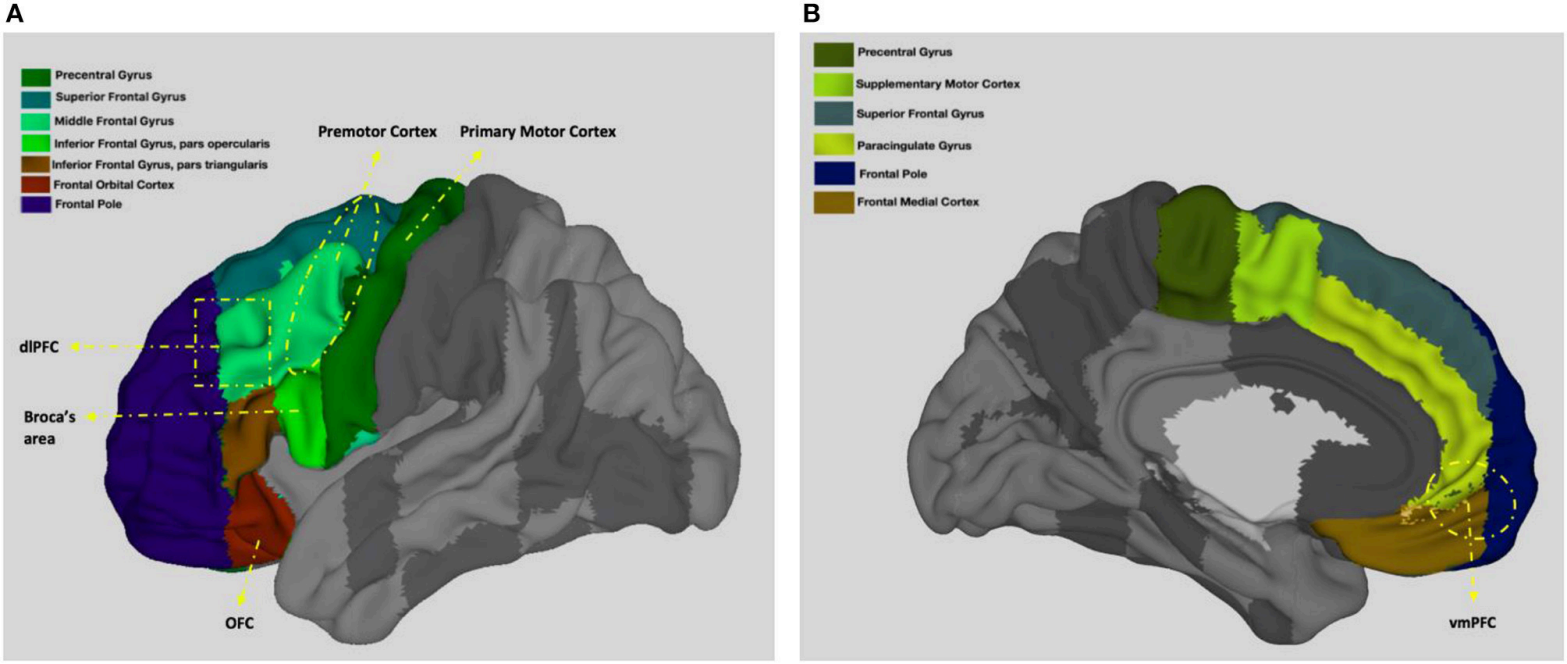
Neuroanatomy of the frontal lobes. **(A)** presents left view and **(B)** presents right view. Adapted from the Harvard-Oxford atlas developed at the Center for Morphometric Analysis (CMA), and distributed with the FMRIB Software Library (FSL) (Bakker et al., 2015), 3D Surface View (Majka et al., 2012).

Among the important functions of the frontal lobes are voluntary movement and control of behavior (Colby and Olson, 2003). Below, I detail the role of the frontal lobes in motor behavior with reference to four key regions with distinct yet interrelated functions (frontal lobes and the location of some of the main frontal lobe areas are shown on Figure 2). While the role of premotor cortex in the mirror neuron network seems to be the most sociologically relevant function on the surface, together these regions enable humans to perform complex motor tasks including speaking and thus it is crucial to have a basic understanding of all of them.

### The Primary Motor Area

The primary motor cortex, which is located on the precentral gyrus, contains a somatotopically organized (point by point correspondence of body parts to the motor areas) map of the muscles of the body that represents the leg medially, situated in the middle, the head laterally, toward the sides, and other body parts at intermediate locations (Colby and Olson, 2003). Neurons in the primary motor cortex receive proprioceptive input from and innervate these muscle groups (Colby and Olson, 2003). The primary motor cortex is the largest contributor to the corticospinal tract carrying action potentials to the spinal cord indicating its significance in muscle movements (Porter and Lemon, 1993; Dum and Strick, 2002). Studies with monkeys reveal that neurons in this area are involved in both selection of the direction of movement and the patterns of muscle activation (Kakei et al., 1999). Various other studies also observe that the primary motor cortex is activated before and during movements, during learned arrest (inhibition or restrain) occurring before movements, or in envisioning movements (Georgopoulos et al., 1982, 1984; Smyrnis et al., 1992; Decety et al., 1994; Rao et al., 1996). Another important feature of this region is the plasticity of the synapses that allow distinguished flexibility in motor behavior in animals with larger forebrains (Sanes and Donoghue, 2000).

### The Premotor Area and Broca's Area

The premotor area lies in front of the primary motor cortex on the lateral surface (Weinrich and Wise, 1982; Wise, 1985; Blumenfeld, 2002). The premotor cortex receives visual and somatosensory information (Fogassi et al., 1996; Graziano et al., 1997) and is involved in visually guided movements and limb positioning in space (Godschalk et al., 1981; Kurata, 1993; Kakei et al., 2001) as well as limb ownership (self-attribution of body parts; Ehrsson et al., 2004). In addition to areas involved in limb movements, the premotor cortex also includes the frontal eye fields, a region that shows increased neuronal activity during the execution of eye movements (Paus, 1996). The frontal eye field, which is extensively connected to extrastriate visual cortex (Schall, 2002), controls saccadic eye movements by shifting eye gaze (Bruce et al., 1985; Schall, 2002) as well as attention (Schall, 2002).

Broca's area, which is located in the inferior frontal gyrus of the dominant sphere (see Figure 2), controls the ability to coordinate the muscles necessary for speaking (Amunts et al., 1999; Waxman, 2010). Named after famous French anatomist Paul Broca due to his studies associating lesions in this area with speech impediments (Keller et al., 2009), Broca's area coordinates vocalizations (Afifi and Bergman, 2005) and is also considered to be specialized in identifying natural principles of language (Musso et al., 2003). Lesions in this area lead to Broca's aphasia, which is characterized as difficulty in motor production of speech (including poor repetition, problems with naming, reading, and writing) while comprehension is intact (Stuss and Benson, 1984). In addition to speaking, it is also demonstrated that Broca's area and its right homolog is involved in musical syntax processing (Maess et al., 2001).

First discovered in monkeys, the so-called mirror neurons in the premotor cortex (F5 area) were neurons firing both when the monkey performed and act and observed another doing a similar act (for example picking up food; Di Pellegrino et al., 1992; Gallese et al., 1996; Rizzolatti et al., 1996). Later studies have confirmed similar activation in the Broca's region in humans when people observed or executed a similar action (Heiser et al., 2003; Rizzolatti and Craighero, 2004; Kilner et al., 2009) though human studies of mirror neurons are much more scant leading to skepticism among some. While initially considered as an action recognition mechanism, later studies have expanded the role of mirror neurons to intention recognition (Iacoboni et al., 2005; Kaplan and Iacoboni, 2006). In this view, the mirror neuron network is involved in detecting the intentions of actors while watching their actions, a building block for empathy (Gallese and Goldman, 1998; Preston and De Waal, 2002; Decety and Jackson, 2004). An important implication of the mirror neuron hypothesis is that the evolved capacities for empathy involve a much more basic and automatic system that has evolved “bottom-up” from simple motor functions for action and perception (Iacoboni, 2009). Furthermore, evidence that mirror neurons also respond to sounds (Kohler et al., 2002; Gazzola et al., 2006) and that the mirror neurons in monkeys are homolog of the Broca's area, responsible for speech production in humans (Petrides et al., 2005), gave way to the hypothesis that mirror neurons might have harbored the evolution of language in humans, bridging “doing” with “communicating” (Rizzolatti and Arbib, 1998).

### The Supplementary Motor Area

The supplementary motor area, which lies in front of the primary motor cortex on the medial surface (Tanji, 1994; Blumenfeld, 2002), is involved in the motor sequencing of limb movements (Roland et al., 1980; Mushiake et al., 1991) and saccades (Gaymard et al., 1990, 1993; Colby and Olson, 2003). Studies show that the supplementary motor area allows complex sequential motor function by the planning and coding of sequences of movements before the movements are executed (Roland et al., 1980; Tanji and Shima, 1994).

### Sociological Implications of the Motor Functions of the Frontal Lobes

At the first level of proximal operation, I propose that the motor activation in the frontal lobes correspond to the most basic capacities for intention reading (or reading other minds) through action perception via the activation of the mirror neuron systems. These motor operations underlie several symbolic interactional processes such as written or spoken language, acts and gestures (Mead, 1934); and thus, serve as the most proximal causal precursor to extending minds beyond the individual, propagating social connectedness. Symbolic social interaction is an interactive process of communication via gesturing (through spoken, written or body language) in which both parties adjust to each other (Mead, 1934; Goffman, 1959). This interaction or exchange process, which can be characterized by voluntary muscle and eye movements as well as motor production of speech all depend, I argue, on the voluntary motor behavior regions of the frontal lobes (e.g., the primary and supplementary motor areas). However, human beings act toward things on the basis of the meanings they assign to them, and these meanings are constructed as well as modified and reconstructed through social interaction (Blumer, 1969).

Dovetailing to Durkheim (1912/1995) and the concept of “collective effervescence”—shared, collective communication or feeling through participating in the same action, neo-Meadian sociological theories of symbolic social interaction, specifically Interaction Rituals theory would particularly benefit from an understanding of the action perception in the motor cortex. According to Interaction Rituals theory, actors engaged in the shared ritual or act generate a collective emotional energy that promotes feelings of solidarity (Collins, 1981, 1987, 1993, 2014; Collins and Hanneman, 1998). Actors in social interaction view this emotional energy and solidarity as an ultimate good and seek further interactions to maximize their collective emotions, hence creating interaction rituals (Collins, 1993). The ways interaction rituals mobilize people or increase community participation have been observed in diverse settings such as megachurches (Wellman Jr et al., 2014), fair trade retail and coffee shops (Brown, 2011) as well as online networks (Maloney, 2013). Based on the literature reviewed in the previous section, neuroscientific research suggests that actions of others are marked and potentially translated into meanings automatically in the motor prefrontal regions through the mirror neuron system. We would expect mirror neuron activation when people are engaging in interaction rituals producing emotional energies. So, one mechanism through which interaction rituals create emotional energy and promote solidarity might be by creating simultaneous shared representations of rituals in the brains of different group members and hence creating a magnifying effect on feelings of participation and inclusion.

## The Prefrontal Cortex and Abstract Functioning

While the motor areas of the frontal lobes provide the infrastructure for connecting self and the others through motor mimicry, the regions in the prefrontal cortex are suggested to be underlying more abstract evaluations of others and empathy (Uddin et al., 2007). The prefrontal cortex (PFC) hosts two important areas related to the more higher-order operational mechanisms that guide behavior including emotion and decision making. The PFC lies anterior to the motor and premotor areas (Afifi and Bergman, 2005). The prefrontal cortex, which is highly developed in primates, especially in humans, is the largest part of the frontal lobes and phylogenetically one of the latest developing areas (Jerison, 1994; Fuster, 2001). The PFC includes the higher order heteromodal association cortex employed in abstract reasoning, judgment, and social behavior (Blumenfeld, 2002; Waxman, 2010). The PFC has bidirectional connections with various other cortical (such as association cortices of other lobes, and the limbic lobe) and subcortical (including hypothalamus, thalamus, amygdala, and hippocampal formation) areas (Fuster, 2001; Blumenfeld, 2002; Afifi and Bergman, 2005).

Many valuable insights about the functions of the prefrontal cortex have been gained by studying the patients with damage to the prefrontal cortex. Among the most famous and the earliest documented of these is the case of Phineas Gage—a railroad construction worker in New England whose forehead was penetrated by a thick iron bar. After damage to his prefrontal cortex, Phineas Gage experienced immense personality and behavioral changes (Damasio et al., 1994). While the extent and the precise location as well as the exact nature of the personality and behavioral changes in the case of Phineas Gage cannot be know with certainty (Macmillan, 2000), this case has become a symbolic marker in the study of neuroscience, pointing to the importance of the prefrontal cortex in abstract functioning including emotional and executive processes. More recent case studies of lesion patients with damage to the PFC also demonstrate the significance of this area in decision-making, social behavior, and emotion (Damasio et al., 1990; Bechara et al., 1996, 2000; Anderson et al., 2000, 2006). For example, patients that had damage to their prefrontal cortex early in their lives, such as in infancy, have abnormal development of social and moral behavior independent of their social environment (Anderson et al., 1999). They disregard social and moral standards, do not express guilt or remorse, and show consistent irresponsibility.

In this paper, I suggest a hierarchical division of labor between two primary prefrontal cortex regions: (a) the ventromedial prefrontal cortex (vmPFC) (involved in emotional regulation and motivation) and (b) the dorsolateral prefrontal cortex (dlPFC) (responsible for top-down inhibition and executive functions). This functional division, I suggest, also has implications for sociological research, with vmPFC corresponding to empathy, Theory of Mind, and intuitive moral judgments and dlPFC regulating top-down control of these empathy and inter-group processes that I describe in more detail in the section Sociological Implications of the More Abstract Functions of the Prefrontal Cortices: Emotional Regulation and Self-reflexivity. Below, I illustrate the role of the PFC in motivation and emotion and executive function by providing empirical evidence.

### Motivation and Emotional Regulation

The ventromedial regions of the prefrontal cortex, including the orbital PFC, are considered to be important centers for motivation and emotional regulation (Damasio, 1994; Anderson et al., 2006). These regions are medially placed in the frontal region of the prefrontal cortex and are reciprocally connected to sensory cortices and limbic structures (Damasio, 1996; Price, 1999; Berridge and Kringelbach, 2008). While many studies have linked the ventromedial PFC (vmPFC) to emotional regulation (see Adolphs, 2009 for a review), the lower-most orbital frontal parts of the ventromedial PFC region have a more specialized role in reward conditioning, pleasure and happiness (Kringelbach and Berridge, 2009). The orbital PFC activation is correlated with the subjective ratings of the pleasantness of odors (no activation by the unpleasant odors; Rolls et al., 2003), subjective ratings of the pleasantness of water in a thirst experiment (De Araujo et al., 2003), increasing consonance of music (which covaried with subjective pleasantness; Blood and Zatorre, 2001), and perceived attractiveness of faces (O'Doherty et al., 2003).

Among the most emblematic modern examples illustrating the importance of the ventral and orbital PFC in emotional regulation is the case of patient EVR who had tumor bilaterally growing in the orbital and ventral PFC at the age of 35 (Eslinger and Damasio, 1985; Damasio, 1994). After the removal of the tumor with surgery, EVR experienced severe changes in his personal and social life including losing his job, going bankrupt and getting divorced twice, despite his intellectual abilities remaining intact (Eslinger and Damasio, 1985; Saver and Damasio, 1991). Group studies of patients with damage to the same areas also found that these patients had blunt affect, deterioration in goal-directed behavior, seemed to get easily frustrated, showed inappropriate social behavior, and were unable to apprehend that these changes were occurring (Barrash et al., 2000). Other studies with adults with vmPFC damage also found that these patients failed to show autonomic responses to socially meaningful stimuli (Damasio et al., 1990), and failed to avoid disadvantageous choices (Bechara et al., 2000). Damasio et al. (1990) argue that these behavioral abruptions caused by orbital and ventral PFC damage in adult life lead to the behavioral syndrome that they term “acquired sociopathy” due to its close resemblance to the sociopathic disorder (American Psychiatric Association, DSM-III, 1980, currently referred to as antisocial personality disorder in American Psychiatric Association, DSM 5, 2013) caused by genetic and/or environmental factors earlier in life. Cases of early-onset vmPFC damage (during early childhood) demonstrate even more severe social conduct problems such that these patients show a lack of concern and responsibility, criminal tendencies, and seem to be devoid of moral emotions such as remorse or guilt and are unaware of these problems while most other intellectual and cognitive abilities are intact (Anderson et al., 1999, 2000). The severity of the early-onset vmPFC patients' behavioral impairments are thought to be related to their impaired acquisition of social and moral knowledge during development (Anderson et al., 2000).

These results provide support to a prominent theory about the role of emotions in decision-making: the Somatic Marker Hypothesis (Damasio, 1994, 1996). In this theory, the vmPFC is suggested to function as a convergence zone that holds dispositional linkages between factual information about given situations and somato-sensory states (including emotions) through a combination of prior experience and future anticipation (Damasio, 1994, 1996; Damasio and Everitt, 1996). Accordingly, individuals rely on bodily responses (somatic markers) to differentiate among possible options available while making decisions. Our bodies generate responses (preferable vs. not) through a combination of prior experience and future anticipation. If initial positive experience with a stimulus leads to a pleasurable somatic state, our bodies record this state by generating somatic markers. Thus, in a future situation, with the possibility of the engagement with same stimulus, through activation of these somatic markers (even when the positive outcome is absent) our bodies would bias our preferences toward that stimulus (over another). These markers express themselves in emotions and influence our value-relevant decisions; and thus instead of logically deducing appropriate decisions, Damasio suggests that our body “tells” us which options “feels” the most appropriate (Damasio, 1994, 1996).

### Executive Function

Executive functions usually refer to cognitive functions that enable individuals to solve difficult, novel and complex tasks by selecting and integrating actions or thoughts with internal goals and mediating actions across time (MacDonald et al., 2000; Fuster, 2001; Miller and Cohen, 2001). These functions include inhibition, switching, working memory, selective attention, problem solving and organizational skills (Alvarez and Emory, 2006). Various studies indicate that the prefrontal cortex has a critical role in executive function. Patients with frontal lobe lesions perform worse than normal subjects or subjects with damage to other brain areas (see Stuss and Benson, 1984 for a review) in the most commonly employed executive function tests (Alvarez and Emory, 2006) like the Wisconsin Card Sorting Test, Phonemic Verbal Fluency, and Stroop Color Word Interference Test.

While non-frontal as well as different parts of the frontal lobes also contribute to the executive functions (Alvarez and Emory, 2006), these functions are often attributed to the dorsolateral region of the prefrontal cortex (MacDonald et al., 2000). The dorsolateral PFC is not only linked to the occipital, temporal and parietal cortices and receives visual, somatosensory, and auditory information from them but is also closely connected with the motor areas of the brain and is therefore thought to control behavior via these connections (Miller and Cohen, 2001). Involvement in working memory is considered among the important attributes of the dorsolateral PFC (Fuster, 2000; MacDonald et al., 2000). For example, dorsolateral PFC activation is observed when participants asked to hold increasingly longer sequences of items in their memory, when they are prompted to perform multiple tasks (compared to one task at a time; D'esposito et al., 1995; Cohen et al., 1997; Courtney et al., 1998). These findings indicate the importance of the dorsolateral PFC in planning and goal directed behavior since working memory is required in holding goals in mind and achieving it gradually.

Patients with dorsolateral PFC lesions are often observed to be indifferent, abulic or apathetic and show inability to plan ahead, unable to generate hypotheses, have trouble in tasks demanding flexibly shifting sets or changing tasks, have poor verbal fluency, and poor organizational and constructional strategies in learning new tasks (Milner, 1963; Benton, 1968; Jones-Gotman and Milner, 1977; Stuss and Benson, 1984; Waxman, 2010). Alongside general executive function studies, some studies also show that dorsolateral PFC has a role in a cognitive control mechanism related to racial bias inhibition (Stanley et al., 2008).

### Sociological Implications of the More Abstract Functions of the Prefrontal Cortices: Emotional Regulation and Self-Reflexivity

Beyond a precursory mirroring mechanism to empathy, the functional organization of the prefrontal cortex into the emotional and control centers for social behavior offer direct connections with sociological knowledge in three ways: (a) that human moral cognition and empathy is a universal capacity (so several social group biases including racial bias are neither hard-wired nor immalleable), (b) despite this universal, automatic empathic capacity, human empathy is still selective, favoring certain groups over others due to the highly socially susceptible nature of the brain, which restructures activation patterns in response to social pressures, and (c) through reflexive thinking and conscious overwriting, it is possible to top-down control selective empathy and group biases. Below I explain these connections in more detail.

The neuroscience literature on empathy—“reading” or understanding others' intentions, thoughts and emotions—has revealed that this capacity is a human universal underlined by activation in the medial and orbitofrontal prefrontal cortices (alongside temporo-parietal regions of the brain; Frith and Frith, 2001; Saxe et al., 2004; Hynes et al., 2006; Singer, 2006; Shamay-Tsoory et al., 2010; Schurz et al., 2014). Also referred to as the “theory of mind” (ToM), humans' ability to attribute intentions and motivations to others' behaviors, thoughts and desires emerge as young as 4 years old and is severely impaired in children with autism (Frith and Frith, 1999; Gallagher and Frith, 2003; also see Hopcroft, 2013 for a sociological treatise). Traditionally measured through a “false-belief task” that presents a child with a story about an actor who leaves an object in a room (e.g., a piece of chocolate left on the table) and exits the room, after which the location of the object is changed by someone else (e.g., chocolate is put in the drawer) and asks where the returning actor would search for their object (on the table—actor's perspective, or in the drawer—the child's perspective; Wimmer and Perner, 1983). While the age in which this capacity was developed was challenged by some studies adapting a deceptive hiding task in favor of a younger age (age of 3 vs. 4) for ToM development (Chandler et al., 1989; Hala et al., 1991; Hala and Russell, 2001), a meta-analytical study of previous research has confirmed the age of 4 as a critical development stage across cultures for ToM development (Wellman et al., 2001). However, further research has demonstrated that individual variability, especially along language abilities (such as syntax, vocabulary, or semantics) is a strong predictor of ToM differences in children (Milligan et al., 2007). Another study even suggested that language may have a causal role in ToM development as older deaf Nicaraguan adults who have had little language exposure (and have incomplete language knowledge), due to the somewhat recent emergence of the Nicaraguan Sign Language, consistently failed ToM tasks, while younger deaf Nicaraguan adults who have had exposure to the language since childhood did not (Pyers and Senghas, 2009). These studies suggest that while developmental stages are critical in ToM and empathy development, social interaction and context also are crucial.

Moreover, while the capacity for empathy is shared by all humans, neuroscientific research reveals that human empathy is socially selective, favoring certain groups over others (Cikara and Fiske, 2011; Cikara et al., 2011), making this cognitive phenomenon especially relevant for sociology and the study of group processes. The frontal lobe areas involved in self-referential processing and empathy, particularly orbital and medial prefrontal cortices have been shown to respond more to in-group members and friends (Volz et al., 2009; Freeman et al., 2010; Krienen et al., 2010) and showed reduced activity in response to dehumanized and stigmatized others (Harris and Fiske, 2006). These findings fit well with the sociological understanding that symbolic racial boundaries are shaped by cultural resources available to individuals including historical national and religious traditions, education systems and media, and the structural conditions they are placed in such as market positions, social networks, level of criminality in the communities etc. (Lamont, 1992, 2000). Through our involvement in a wide range of groups (such as recreational groups, ethnic groups, and professional groups), we constantly produce and re-establish competing boundaries (Lamont, 1992).

The literature on our selective empathic capacity also aligns well with the current neuroscientific perspectives on inter-group evaluations and bias, which have predominantly been studied with respect to racial attitudes. Racial attitudes and stereotypes have been an important topic for social neurosciences for the last two decades. While majority of early studies have focused on the subcortical structures that are related to basic “fight or flight” responses like the amygdala (e.g., Hart et al., 2000; Phelps et al., 2000; Cunningham et al., 2004; Lieberman et al., 2005; Ronquillo et al., 2007), more recent research has also clearly demonstrated the importance of the frontal lobes for dehumanization and bias inhibition processes involved in prejudice Dehumanization is a cognitive bias that involves the act or psychological process of reducing a human to a non-human being by devoid of mentalizing (or empathizing; Fiske, 2009; see Haslam, 2006 for a review). Often targeting extremely stigmatized out-group members, dehumanization is related to feelings of basic disgust in contrast to feelings of social and moral emotions (Harris and Fiske, 2007; Fiske, 2009). Taking a social neuroscience approach, dehumanization research reports reduced activation in the frontal lobes related to empathy and moral emotions (i.e., medial prefrontal cortex) when dehumanization happens (Harris and Fiske, 2006, 2007; Firat, 2013; Firat et al., 2017). Again, it is important to note that the frontal lobes are not the only brain regions involved in moral judgments or empathy. For example, in a ground breaking study, Young et al. (2010) show that when neural activity in right temporoparietal junction is disrupted with transcranial magnetic stimulation, subjects failed to evaluate harmful intentions of others.

Yet, these automatic processes of selective empathy or inter-group bias can be inhibited and overwritten with deliberate thinking through dlPFC activation, the key center for executive functions including temporal-sequencing, planning, working memory and control. Akin to the capacity for reflexivity (the mind's ability to reflect upon itself) lying at the heart of symbolic social interaction theory (Blumer, 1969), the dorsolateral prefrontal cortex, I suggest, provides flexibility (adaptability to changing stimuli) and cognitive control (inhibiting impulses and regulating coherent, consistent behavior) in continuous interactions. As the highest-order, least proximate functioning mechanism of the frontal lobes, the dorsolateral prefrontal cortices produce reflexive input and regulation for the progress and adaptation of human moral actions. For example, Knutson et al. (2007) showed that dorsolateral PFC is activated when subjects are prompted to categorize stimuli in a way that counter their implicit attitudes regarding race or gender. Similarly, Richeson et al. (2003) found increased dorsolateral PFC activation in individuals who scored higher on measures of racial bias when they are exposed to racial out-group faces, which they interpret as individuals with higher racial bias showing more effort and executive control to reduce their bias (see also Kubota et al. (2012) and Ito and Bartholow (2009) for reviews of neuroscience of racial attitudes and functions of the dlPFC).

The top-down inhibitory role of the dlPFC, especially in racial bias, potentially challenges many mainstream social psychological views that often rely on the implicit yet often deep-seated assumption that the automatic categorization of persons into racial categories is hard-wired or unavoidable (e.g., Fiske, 2002; Dovidio et al., 2010, also evident in many influential social psychological theories like Expectation States Theory and Social Identity Theory, Tafjel and Turner, 1986; Berger and Webster, 2006; Correll and Ridgeway, 2006). Rooted in an understanding of the evolved cognitive capacity for categorical thinking, in this view, humans categorize everything (objects, animals etc.) into groups or natural types based on perceived similarities (Rothbart and Taylor, 1992; Hirschfeld, 1996; GilWhite et al., 2001) because of its adaptive nature for group survival. However, an alternative view from evolutionary anthropology is that races are not natural types and have not divided tribal societies (Kurzban et al., 2001). Racial categorization and encoding is the byproduct of an essentially moral capacity of coalition building; and, race has become a perceptual cue for detecting alliances under historical conditions that created racially un-egalitarian societies (Kurzban et al., 2001; Cosmides et al., 2003). Coalitional alliance differences along racial lines are further perpetuated in modern societies magnifying health and well-being disparities among others (Boyer et al., 2015; Firat and Boyer, 2015). These arguments also fit well with the sociological racial formation theory, which emphasizes that race is constructed through the historical social, political, and economic forces to sustain stratified social relations and cultural dominance (Omi and Winant, 1994).

In sum, observing that the key brain regions involved in processing/regulating socially and morally salient information (such as the vmPFC and the dlPFC) are also involved in racial boundary making would buttress argument that (a) on the one hand, racial bias and categorization are not inevitable (Cosmides et al., 2003; see also Firat et al., 2017), and (b) on the other hand, societal-level ideological and structural changes are required to remove the use of race as a coalitional cue, diminishing racism at the individual-level.

## Future Directions and Research Questions

A more in-depth understanding of the functions and interconnections of the regions of the frontal lobes lead to several future research questions with significant sociological implications. For example, motor regions of the frontal lobes are involved (in coordination with subcortical structures) in automatic learning, habit, and implicit processes (Sanes, 2003). On the one hand, motor learning and automaticity can be altered through explicit information processing via the dorsolateral prefrontal cortices; while, on the other hand, practice or syncopation related changes can also alter motor behavior only through activation of the motor regions (Sanes, 2003; Ashby et al., 2010). Some interesting questions for social scientists and social psychologists would be: Are there two dissociable mechanisms for altering implicit social information processing (e.g., implicit racial bias, emotional reactions, moral intuitions), one through top-down explicit information and another more implicit via other sensory cues or practice? If yes, are they equally effective in altering implicit evaluations? Could one over the other produce more long-lasting strategies for reducing implicit biases? All of these future research questions and directions have the potential to open particularly fruitful avenues of collaboration between sociologists and neuroscientists that will hopefully contribute to our advancement of the complex social behavior.

This theoretical linkage between interaction rituals and the mirror neuron system would also pose several new research questions such as: Do brain correlates of both ritualistic and non-ritualistic interactions recruit mirror neurons? Does the size of the groups in which interaction rituals occur affect mirror neuron network activation, or in other words, would rituals that happen in very large groups (e.g., mass public protests) vs. small groups (e.g., friend circles) be represented the same way in the brain? And, with the increasing online networking technologies, would online interaction rituals be represented the same way as face to face interactions in the brain?

For a moral capacity that encompasses also prosociality and altruism, the ventromedial prefrontal cortices (moral emotional processing or tagging centers of the brain) seem to be more crucial than slower cognitive centers. If emotions are primary in moral empathy and social connectedness, would that mean that in order to promote inter-group solidarity, we can target emotional messaging and empathy? How would these processes differ in the so-called collectivistic vs. individualistic cultures?

As decades of sociological research shows emotions are physiologically experienced yet culturally constructed processes (Thoits, 1989); they are intrinsically linked to cultural norms (Hochschild, 1983; Hochshild, 1989) as well as power and status hierarchies (Kemper, 1981, 1986, 1991) and therefore are important mechanisms in interpersonal evaluations (Smith-Lovin, 1990; Robinson and Smith-Lovin, 2006), social exchange processes (Lawler et al., 2000; Lawler, 2001) as well as in self and identity processes (Burke, 1991, 2008). How would all these structural and cultural variations shape, alter, challenge emotive processes, and activation differences in the ventromedial prefrontal cortices? Notwithstanding, several research studies point that the vmPFC and the dlPFC, the emotional and cognitive hubs of the prefrontal cortices, are also reciprocally connected, regulating self-control, reward delay and impulse control (e.g., Hare et al., 2014; Steinbeis et al., 2014), with potentially dissociable roles in reward processing and value attributions—with dlPFC being more selective for the variability (i.e., high vs. low) of values (Kahnt et al., 2011). One research study even demonstrated that damage to the dorsolateral but not ventromedial prefrontal cortices diminished monetary contributions in a public goods game (Wills et al., 2018). This body of research might indicate that while a more holistic theory of morality and social cooperation should take into account both emotional and cognitive components of neural coding, more cognitive dlPFC activation perhaps could be involved in comparing various social markers including social status, economic status or even cultural markers of power.

## Summary

In summary, the frontal lobes have a variety of diverse and complicated functions that I divide under three primary conceptual categories with specific implications for social organization and connectedness: a. control of voluntary behavior (mirror neuron and action perception processes), b. emotional and motivational regulation (Theory of Mind and emotional empathy), and c. executive functioning (top-down regulation of selective empathy and racial attitudes). I should note that these functional categories naturally overlap with each other (e.g., influence of emotions on attention) and often are interdependent. Moreover, even though they are mainly attributed to the frontal lobes by previous research, many other cortical and subcortical regions also carry out important roles in these functions (Alvarez and Emory, 2006). I argue that by performing these functions in a hierarchical manner, the frontal lobes provide a specially organized infrastructure for human social connectedness that range from the most proximate and basic capacity for intention reading and mirroring to more complex emotional humanization/dehumanization and abstract top-down regulation of inter-group processes.

## Concluding Remarks

To conclude, this paper attempts to outline the implications of the frontal lobe functions for both the micro-individual and the macro-society. Our biology is fundamentally social, meaning that “it is our nature to nurture and to be nurtured” (Wexler, 2006, p. 13). Among the animal species, the human brain has the longest period in which its growth is shaped by the environment; however, the human brain is not only shaped by the environment, it also shapes the social environment (Wexler, 2006). The frontal lobes enable humans (as one of the species with the largest frontal lobes) to engage in complex, goal-directed behaviors consistent, and continuous across time, yet also flexible and responsive to the stimuli and changes both external and internal to the individual. Many of the frontal lobe functions underlie sophisticated behaviors that are core for complex social organization and life, such as social and symbolic interaction, moral cognition and behavior as well as empathy and inter-group attitudes. The frontal lobes, therefore, help sustain us not only as individuals but also as social systems and societies. However, this is not to propose that the frontal lobes or the brain are independent causal agents in human social functioning (a biologically reductionist view often attributed to neurosciences). My point is rather to explain the inner-mechanisms of a complex, social organism that can help us better understand how environmental factors (both evolutionarily and developmentally) shape our biology, which in turn affects social behavior. This dynamic reciprocal interaction is among the hallmark of our social life, and is thus of crucial importance to sociology.

## Author Contributions

The author confirms being the sole contributor of this work and has approved it for publication.

### Conflict of Interest Statement

The author declares that the research was conducted in the absence of any commercial or financial relationships that could be construed as a potential conflict of interest.

## References

[B1] Abend G. (2011). Thick concepts and the moral brain. Eur. J. Sociol. Arch. Européennes de Sociologie 52, 143–172. 10.1017/S0003975611000051

[B2] Abend G. (2017). What are neural correlates neural correlates of? BioSocieties 12, 415–438. 10.1057/s41292-016-0019-y

[B3] Abend G. Petre C. Sauder M. (2013). Styles of causal thought: an empirical investigation. Am. J. Sociol. 119, 602–654. 10.1086/675892

[B4] Adolphs R. (2009). The social brain: neural basis of social knowledge. Annu. Rev. Psychol. 60, 693–716. 10.1146/annurev.psych.60.110707.163514 18771388 PMC2588649

[B5] Afifi A. K. Bergman R. A. (2005). Functional Neuroanatomy: Text and Atlas, 2nd Edn. New York, NY: McGraw-Hill.

[B6] Alvarez J. A. Emory E. (2006). Executive function and the frontal lobes: a meta-analytic review. Neuropsychol. Rev. 16, 17–42. 10.1007/s11065-006-9002-x 16794878

[B7] American Psychiatric Association (1980). Diagnostic and Statistical Manual of Mental Disorders, 3rd Edn. Washington, DC: American Psychiatric Association.

[B8] American Psychiatric Association (2013). Diagnostic and Statistical Manual of Mental Disorders, 5th Edn. Washington, DC: American Psychiatric Association.

[B9] Amunts K. Schleicher A. Bürgel U. Mohlberg H. Uylings H. B. Zilles K. (1999). Broca's region revisited: cytoarchitecture and intersubject variability. J. Comp. Neurol. 412, 319–341. 10441759 10.1002/(sici)1096-9861(19990920)412:2<319::aid-cne10>3.0.co;2-7

[B10] Anderson S. W. Barrash J. Bechara A. Tranel D. (2006). Impairments of emotion and real-world complex behavior following childhood-or adult-onset damage to ventromedial prefrontal cortex. J. Int. Neuropsychol. Soc. 12, 224–235. 10.1017/S1355617706060346 16573856

[B11] Anderson S. W. Bechara A. Damasio H. Tranel D. Damasio A. R. (1999). Impairment of social and moral behavior related to early damage in human prefrontal cortex. Nat. Neurosci. 2, 1032–1037. 10.1038/14833 10526345

[B12] Anderson S. W. Damasio H. Jones R. D. Tranel D. (1991). Wisconsin Card Sorting Test performance as a measure of frontal lobe damage. J. Clin. Exp. Neuropsychol. 13, 909–922. 10.1080/01688639108405107 1779030

[B13] Anderson S. W. Damasio H. Tranel D. Damasio A. R. (2000). Long-term sequelae of prefrontal cortex damage acquired in early childhood. Dev. Neuropsychol. 18, 281–296. 10.1207/S1532694202Anderson 11385828

[B14] Ashby F. G. Turner B. O. Horvitz J. C. (2010). Cortical and basal ganglia contributions to habit learning and automaticity. Trends Cogn. Sci. 14, 208–215. 10.1016/j.tics.2010.02.001 20207189 PMC2862890

[B15] Bakker R. Tiesinga P. Kötter R. (2015). The Scalable Brain Atlas: instant web-based access to public brain atlases and related content. Neuroinformatics 13, 353–66. 10.1007/s12021-014-9258-x 25682754 PMC4469098

[B16] Baronchelli A. Ferrer-i-Cancho R. Pastor-Satorras R. Chater N. Christiansen M. H. (2013). Networks in cognitive science. Trends Cogn. Sci. 17, 348–360. 10.1016/j.tics.2013.04.010 23726319

[B17] Barrash J. Tranel D. Anderson S. W. (2000). Acquired personality disturbances associated with bilateral damage to the ventromedial prefrontal region. Dev. Neuropsychol. 18, 355–381. 10.1207/S1532694205Barrash 11385830

[B18] Bechara A. Tranel D. Damasio H. (2000). Characterization of the decision-making deficit of patients with ventromedial prefrontal cortex lesions. Brain 123, 2189–2202. 10.1093/brain/123.11.2189 11050020

[B19] Bechara A. Tranel D. Damasio H. Damasio A. R. (1996). Failure to respond autonomically to anticipated future outcomes following damage to prefrontal cortex. Cereb. Cort. 6, 215–225. 10.1093/cercor/6.2.215 8670652

[B20] Benton A. L. (1968). Differential behavioral effects in frontal lobe disease. Neuropsychologia 6, 53–60. 10.1016/0028-3932(68)90038-9

[B21] Berger J. Webster M. Jr. (2006). Expectations, status, and behavior, in Contemporary Social Psychological Theories, eds Burke P. J. (Stanford, CA: Stanford University Press), 268–300.

[B22] Berridge K. C. Kringelbach M. L. (2008). Affective neuroscience of pleasure: reward in humans and animals. Psychopharmacology 199, 457–480. 10.1007/s00213-008-1099-6 18311558 PMC3004012

[B23] Blood A. J. Zatorre R. J. (2001). Intensely pleasurable responses to music correlate with activity in brain regions implicated in reward and emotion. Proc. Natl. Acad. Sci. U.S.A. 98, 11818–11823. 10.1073/pnas.191355898 11573015 PMC58814

[B24] Blumenfeld H. (2002). Neuroanatomy Through Clinical Cases. Sunderland: Sinauer Associates.

[B25] Blumer H. (1969). Symbolic Interactionism. Englewood Cliffs: Prentice-Hall.

[B26] Boyer P. Firat R. van Leeuwen F. (2015). Safety, threat, and stress in intergroup relations: a coalitional index model. Persp. Psychol. Sci. 10, 434–450. 10.1177/1745691615583133 26177946

[B27] Brown K. R. (2011). Interaction ritual chains and the mobilization of conscientious consumers. Qual. Sociol. 34, 121–141. 10.1007/s11133-010-9188-3

[B28] Bruce C. J. Goldberg M. E. Bushnell M. C. Stanton G. B. (1985). Primate frontal eye fields. II. Physiological and anatomical correlates of electrically evoked eye movements. J. Neurophysiol. 54, 714–734. 10.1152/jn.1985.54.3.714 4045546

[B29] Burke P. J. (1991). Identity processes and social stress. Am. Sociol. Rev. 836–849. 10.2307/2096259

[B30] Burke P. J. (2008). Identity, social status, and emotion, in Soc. Struct. Emot., eds Clay-Warner J. Robinson D. (San Diego, CA: Academic Press), 75–93. 10.1016/B978-0-12-374095-3.00005-7

[B31] Chandler M. Fritz A. S. Hala S. (1989). Small-scale deceit: deception as a marker of two-, three-, and four-year-olds' early theories of mind. Child Dev. 1263–1277. 10.2307/1130919 2612240

[B32] Cikara M. Botvinick M. M. Fiske S. T. (2011). Us versus them. Psychol. Sci. 22, 306–313. 10.1177/0956797610397667 21270447 PMC3833634

[B33] Cikara M. Fiske S. T. (2011). Bounded empathy: neural responses to outgroup targets' (mis)fortunes. J. Cogn. Neurosci. 23, 3791–3803. 10.1162/jocn_a_00069 21671744 PMC3792561

[B34] Cohen J. D. Perlstein W. M. Braver T. S. Nystrom L. E. Noll D. C. Jonides J.. (1997). Temporal dynamics of brain activation during a working memory task. Nature 386, 604–608. 10.1038/386604a0 9121583

[B35] Colby C. L. Olson C. R. (2003). Spatial Cognition, in Fundamental Neuroscience, 2nd Edn, eds Squire L. R. Bloom F. E. McConnell S. K. Roberts J. L. Spitzer N. C. Zigmond M. J. (San Diego, CA: Academic Press), 1229–1247.

[B36] Collins R. (1981). On the microfoundations of macrosociology. Am. J. Sociol. 86, 984–1014. 10.1086/227351

[B37] Collins R. (1987). Interaction ritual chains, power and property: The micro-macro connection as an empirically based theoretical problem, in The Micro-Macro Link, eds Alexander J. Giesen B. Munch R. Smelser N. (Berkeley, CA: University of California Press), 193–206.

[B38] Collins R. (1993). Emotional energy as the common denominator of rational action. Rational. Soc. 5, 203–230. 10.1177/1043463193005002005

[B39] Collins R. (2014). Interaction Ritual Chains, Vol. 62. Princeton, NJ: Princeton University Press.

[B40] Collins R. Hanneman R. (1998). Modelling the interaction ritual theory of solidarity, in The Problem of Solidarity: Theories and Models, eds Doreian P. Fararo T. J. (New York, NY: Routledge), 213–237.

[B41] Correll S. J. Ridgeway C. L. (2006). Expectation States Theory. In Handbook of Social Psychology. Boston, MA: Springer. 10.1007/0-387-36921-X_2

[B42] Cosmides L. Tooby J. Kurzban R. (2003). Perceptions of race. Trends Cogn. Sci. 7, 173–179. 10.1016/S1364-6613(03)00057-3 12691766

[B43] Courtney S. M. Petit L. Maisog J. M. Ungerleider L. G. Haxby J. V. (1998). An area specialized for spatial working memory in human frontal cortex. Science 279, 1347–1351. 10.1126/science.279.5355.1347 9478894

[B44] Cunningham W. A. Johnson M. K. Raye C. L. Gatenby J. C. Gore J. C. Banaji M. R. (2004). Separable neural components in the processing of black and white faces. Psychol. Sci. 15, 806–813. 10.1111/j.0956-7976.2004.00760.x 15563325

[B45] Damasio A. R. (1994). Descartes' Error: Emotion, Reason, and the Human Brain. New York, NY: Grosset/Putnam.

[B46] Damasio A. R. (1996). The somatic marker hypothesis and the possible functions of the prefrontal cortex. Phil. Trans. R. Soc. Lond. B 351, 1413–1420. 10.1098/rstb.1996.0125 8941953

[B47] Damasio A. R. Everitt B. J. (1996). the somatic marker hypothesis and the possible functions of the prefrontal cortex [and Discussion]. Philos. Trans. R. Soc. Lond. B. Biol. Sci. 351, 1413–1420. 8941953 10.1098/rstb.1996.0125

[B48] Damasio A. R. Tranel D. Damasio H. (1990). Individuals with sociopathic behavior caused by frontal damage fail to respond autonomically to social stimuli. Behav. Brain Res. 41, 81–94. 10.1016/0166-4328(90)90144-4 2288668

[B49] Damasio H. Grabowski T. Frank R. Galaburda A. M. Damasio A. R. (1994). The return of Phineas Gage: clues about the brain from the skull of a famous patient. Science 264, 1102–1105. 10.1126/science.8178168 8178168

[B50] De Araujo I. E. Rolls E. T. Kringelbach M. L. McGlone F. Phillips N. (2003). Taste-olfactory convergence, and the representation of the pleasantness of flavour, in the human brain. Eur. J. Neurosci. 18, 2059–2068. 10.1046/j.1460-9568.2003.02915.x 14622239

[B51] Decety J. Jackson P. L. (2004). The functional architecture of human empathy. Behav. Cogn. Neurosci. Rev. 3, 71–100. 10.1177/1534582304267187 15537986

[B52] Decety J. Perani D. Jeannerod M. Bettinardi V. Tadary B. Woods R.. (1994). Mapping motor representations with positron emission tomography. Nature 371, 600–602. 10.1038/371600a0 7935791

[B53] D'esposito M. Detre J. A. Alsop D. C. Shin R. K. Atlas S. Grossman M. (1995). The neural basis of the central executive system of working memory. Nature 378, 279–281. 10.1038/378279a0 7477346

[B54] Di Pellegrino G. Fadiga L. Fogassi L. Gallese V. Rizzolatti G. (1992). Understanding motor events: a neurophysiological study. Exp. Brain Res. 91, 176–180. 10.1007/BF00230027 1301372

[B55] Dovidio J. F. Hewstone M. Glick P. Esses V. M. (2010). Prejudice, stereotyping and discrimination: theoretical and empirical overview, in The SAGE Handbook of Prejudice, Stereotyping and Discrimination, eds Dovidio J. F. Hewstone M. Glick P. Esses V. M. (London: SAGE Publications Ltd.), 3–29. 10.4135/9781446200919.n1

[B56] Dum R. P. Strick P. L. (2002). Motor areas in the frontal lobe of the primate. Physiol. Behav. 77, 677–682. 10.1016/S0031-9384(02)00929-0 12527018

[B57] Durkheim E. (1912/1995). The Elementary Forms of the Religious life. New York, NY: Free Press.

[B58] Ehrsson H. H. Spence C. Passingham R. E. (2004). That's my hand! Activity in premotor cortex reflects feeling of ownership of a limb. Science 305, 875–877. 10.1126/science.1097011 15232072

[B59] Eslinger P. J. Damasio A. R. (1985). Severe disturbance of higher cognition after bilateral frontal lobe ablation patient EVR. Neurology 35, 1731–1731. 10.1212/WNL.35.12.1731 4069365

[B60] Firat R. Hitlin S. (2012). Morally bonded and bounded: A sociological introduction to neurology, in Biosociology and Neurosociology (Emerald Group Publishing Limited), eds Kalkhoff W. Thye S. R. Lawler E. J. (Bingley: Emerald Group Publishing Limited), 165–199. 10.1108/S0882-6145(2012)0000029009

[B61] Firat R. McPherson C. M. (2010). Toward an integrated science of morality, in Handbook of the Sociology of Morality, eds Hitlin S. Vaisey S. (New York, NY: Springer), 361–384. 10.1007/978-1-4419-6896-8_19

[B62] Firat R. B. (2013). Apathetic Racism Theory: A Neurosociological Study of How Moral Emotions Perpetuate Inequality. Dissertation thesis, Iowa City, IA: University of Iowa.

[B63] Firat R. B. Boyer P. (2015). Coalitional affiliation as a missing link between ethnic polarization and well-being: an empirical test from the European Social Survey. Soc. Sci. Res. 53, 148–161. 10.1016/j.ssresearch.2015.05.006 26188444

[B64] Firat R. B. Hitlin S. Magnotta V. Tranel D. (2017). Putting race in context: social class modulates processing of race in the ventromedial prefrontal cortex and amygdala. Soc. Cogn. Affect. Neurosci. 12, 1314–1324. 10.1093/scan/nsx052 28398590 PMC5597864

[B65] Fiske S. T. (2002). What we know now about bias and intergroup conflict, the problem of the century. Curr. Dir. Psychol. Sci. 11, 123–128. 10.1111/1467-8721.00183

[B66] Fiske S. T. (2009). From dehumanization and objectification to rehumanization: Neuroimaging studies on the building blocks of empathy. Ann. N. Y. Acad. Sci. 1167, 31–34. 10.1111/j.1749-6632.2009.04544.x 19580549 PMC3777639

[B67] Fogassi L. Gallese V. Fadiga L. Luppino G. Matelli M. Rizzolatti G. (1996). Coding of peripersonal space in inferior premotor cortex (area F4). J. Neurophysiol. 76, 141–157. 10.1152/jn.1996.76.1.141 8836215

[B68] Franks D. D. (2010). Neurosociology: The Nexus Between Neuroscience and Social Psychology. New York, NY: Springer Science and Business Media.

[B69] Franks D. D. Turner J. H. (2013). Handbook of Neurosociology. New York, NY: Springer. 10.1007/978-94-007-4473-8

[B70] Freeman J. B. Schiller D. Rule N. O. Ambady N. (2010). The neural origins of superficial and individuated judgments about ingroup and outgroup members. Hum. Brain Mapp. 31, 150–159. 10.1002/hbm.20852 19618409 PMC6870618

[B71] Frith C. Frith U. (1999). Interacting minds A biological basis. Science 286, 1692–1695. 10.1126/science.286.5445.1692 10576727

[B72] Frith U. Frith C. (2001). The biological basis of social interaction. Curr. Dir. Psychol. Sci. 10, 151–155. 10.1111/1467-8721.00137

[B73] Fuster J. M. (2000). Executive frontal functions. Exp. Brain Res. 133, 66–70. 10.1007/s002210000401 10933211

[B74] Fuster J. M. (2001). The prefrontal cortex—an update: time is of the essence. Neuron 30, 319–333. 10.1016/S0896-6273(01)00285-9 11394996

[B75] Fuster J. M. (2002). Frontal lobe and cognitive development. J. Neurocytol. 31, 373–385. 10.1023/A:1024190429920 12815254

[B76] Gallagher H. L. Frith C. D. (2003). Functional imaging of ‘theory of mind'. Trends Cogn. Sci. 7, 77–83. 10.1016/S1364-6613(02)00025-6 12584026

[B77] Gallese V. (2001). The ‘shared manifold' hypothesis. From mirror neurons to empathy. J. Conscious. Stud. 8, 33–50.

[B78] Gallese V. Fadiga L. Fogassi L. Rizzolatti G. (1996). Action recognition in the premotor cortex. Brain 119, 593–609. 10.1093/brain/119.2.593 8800951

[B79] Gallese V. Goldman A. (1998). Mirror neurons and the simulation theory of mind-reading. Trends Cogn. Sci. 2, 493–501. 10.1016/S1364-6613(98)01262-5 21227300

[B80] Gaymard B. Pierrot-Deseilligny C. Rivaud S. (1990). Impairment of sequences of memory-guided saccades after supplementary motor area lesions. Ann. Neurol. 28, 622–626. 10.1002/ana.410280504 2260848

[B81] Gaymard B. Rivaud S. Pierrot-Deseilligny C. (1993). Role of the left and right supplementary motor areas in memory-guided saccade sequences. Ann. Neurol. 34, 404–406. 10.1002/ana.410340317 8363358

[B82] Gazzola V. Aziz-Zadeh L. Keysers C. (2006). Empathy and the somatotopic auditory mirror system in humans. Curr. Biol. 16, 1824–1829. 10.1016/j.cub.2006.07.072 16979560

[B83] Georgopoulos A. P. Caminiti R. Kalaska J. F. (1984). Static spatial effects in motor cortex and area 5: quantitative relations in a two-dimensional space. Exp. Brain Res. 54, 446–454. 10.1007/BF00235470 6723864

[B84] Georgopoulos A. P. Kalaska J. F. Caminiti R. Massey J. T. (1982). On the relations between the direction of two-dimensional arm movements and cell discharge in primate motor cortex. J. Neurosci. 2, 1527–1537. 10.1523/JNEUROSCI.02-11-01527.1982 7143039 PMC6564361

[B85] GilWhite F. Astuti R. Atran S. Banton M. Boyer P. Gelman S. A.. (2001). Are ethnic groups biological species to the human brain? Essentialism in our cognition of some social categories. Curr. Anthropol. 42, 515–553. 10.1086/321802

[B86] Godschalk M. Lemon R. N. Nijs H. G. T. Kuypers H. G. J. M. (1981). Behaviour of neurons in monkey peri-arcuate and precentral cortex before and during visually guided arm and hand movements. Exp. Brain Res. 44, 113–116. 10.1007/BF00238755 7274360

[B87] Goffman E. (1959). The Presentation of Self in Everyday Life. Garden City: Anchor.

[B88] Graziano M. S. Hu X. T. Gross C. G. (1997). Visuospatial properties of ventral premotor cortex. J. Neurophysiol. 77, 2268–2292. 10.1152/jn.1997.77.5.2268 9163357

[B89] Greene J. D. Sommerville R. B. Nystrom L. E. Darley J. M. Cohen J. D. (2001). An fMRI investigation of emotional engagement in moral judgment. Science 293, 2105–2108. 10.1126/science.1062872 11557895

[B90] Hala S. Chandler M. Fritz A. S. (1991). Fledgling theories of mind: Deception as a marker of three-year-olds' understanding of false belief. Child Dev. 62, 83–97. 10.2307/1130706

[B91] Hala S. Russell J. (2001). Executive control within strategic deception: A window on early cognitive development?. J. Exp. Child Psychol. 80, 112–141. 10.1006/jecp.2000.2627 11529671

[B92] Hare T. A. Hakimi S. Rangel A. (2014). Activity in dlPFC and its effective connectivity to vmPFC are associated with temporal discounting. Front. Neurosci. 8:50. 10.3389/fnins.2014.00050 24672421 PMC3957025

[B93] Harris L. T. Fiske S. T. (2006). Dehumanizing the lowest of the low: Neuroimaging responses to extreme out-groups. Psychol. Sci. 17, 847–853. 17100784 10.1111/j.1467-9280.2006.01793.x

[B94] Harris L. T. Fiske S. T. (2007). Social groups that elicit disgust are differentially processed in mPFC. Soc. Cogn. Affect. Neurosci. 2, 45–51. 10.1093/scan/nsl037 18985118 PMC2555430

[B95] Hart A. J. Whalen C. A. P. J. Shin L. M. McInerney S. C. Fischer H. Rauch S. L. (2000). Differential response in the human amygdala to racial outgroup vs ingroup face stimuli. Neuroreport 11, 2351–2355. 10.1097/00001756-200008030-00004 10943684

[B96] Haslam N. (2006). Dehumanization: an integrative review. Pers. Soc. Psychol. Rev. 10, 252–264. 10.1207/s15327957pspr1003_4 16859440

[B97] Heiser M. Iacoboni M. Maeda F. Marcus J. Mazziotta J. C. (2003). The essential role of Broca's area in imitation. Eur. J. Neurosci. 17, 1123–1128. 10.1046/j.1460-9568.2003.02530.x 12653990

[B98] Hirschfeld L. A. (1996). Race in the Making: Cognition, Culture, and the Child's Construction of Human Kinds. Cambridge: The MIT Press.

[B99] Hochschild A. R. (1983). The Managed Heart: Commercialization of Human Feeling. Berkeley: University of California Press.

[B100] Hochshild A. (1989). The Second Shift: Working Parents and the Revolution at Home. New York, NY: Viking.

[B101] Hopcroft R. L. (2013). Neurosociology and theory of mind (TOM), in Handbook of Neurosociology (Dordrecht: Springer), 231–241. 10.1007/978-94-007-4473-8_16

[B102] Hopcroft R. L. (2016). Grand challenges in evolutionary Sociology and Biosociology. Front. Soc. 1:2. 10.3389/fsoc.2016.00002

[B103] Hynes C. A. Baird A. A. Grafton S. T. (2006). Differential role of the orbital frontal lobe in emotional versus cognitive perspective-taking. Neuropsychologia 44, 374–383. 10.1016/j.neuropsychologia.2005.06.011 16112148

[B104] Iacoboni M. (2009). Imitation, empathy, and mirror neurons. Annu. Rev. Psychol. 60, 653–670. 10.1146/annurev.psych.60.110707.163604 18793090

[B105] Iacoboni M. Molnar-Szakacs I. Gallese V. Buccino G. Mazziotta J. C. Rizzolatti G. (2005). Grasping the intentions of others with one's own mirror neuron system. PLoS Biol. 3:e79. 10.1371/journal.pbio.0030079 15736981 PMC1044835

[B106] Ito T. A. Bartholow B. D. (2009). The neural correlates of race. Trends Cogn. Sci. 13, 524–531. 10.1016/j.tics.2009.10.002 19896410 PMC2796452

[B107] Jerison H. J. (1994). Evolution of the brain, in Neuropsychology, ed D. W. Zaidel (San Diego, CA: Academic Press, Inc.), 53–81. 10.1016/B978-0-08-092668-1.50009-0

[B108] Jones-Gotman M. Milner B. (1977). Design fluency: the invention of nonsense drawings after focal cortical lesions. Neuropsychologia 15, 653–674. 10.1016/0028-3932(77)90070-7 896022

[B109] Kahnt T. Heinzle J. Park S. Q. Haynes J. D. (2011). Decoding different roles for vmPFC and dlPFC in multi-attribute decision making. Neuroimage 56, 709–715. 10.1016/j.neuroimage.2010.05.058 20510371

[B110] Kakei S. Hoffman D. S. Strick P. L. (1999). Muscle and movement representations in the primary motor cortex. Science 285, 2136–2139. 10.1126/science.285.5436.2136 10497133

[B111] Kakei S. Hoffman D. S. Strick P. L. (2001). Direction of action is represented in the ventral premotor cortex. Nat. Neurosci. 4, 1020–1025. 10.1038/nn726 11547338

[B112] Kalkhoff W. Serpe R. T. Pollock J. Miller B. Pfeiffer M. (2016a). Neural processing of identity-relevant feedback, *New Directions in Identity Theory and Research*, eds Stets J. Serpe R. (Oxford: Oxford University Press), 195–238. 10.1093/acprof:oso/9780190457532.003.0008

[B113] Kalkhoff W. Thye S. R. Pollock J. (2016b). Developments in neurosociology. Soc. Comp. 10, 242–258. 10.1111/soc4.12355

[B114] Kaplan J. T. Iacoboni M. (2006). Getting a grip on other minds: Mirror neurons, intention understanding, and cognitive empathy. Soc. Neurosci. 1, 175–183. 10.1080/17470910600985605 18633786

[B115] Keller S. S. Crow T. Foundas A. Amunts K. Roberts N. (2009). Broca's area: nomenclature, anatomy, typology and asymmetry. Brain Lang. 109, 29–48. 10.1016/j.bandl.2008.11.005 19155059

[B116] Kemper T. D. (1981). Social constructionist and positivist approaches to the sociology of emotions. Am. J. Sociol. 87, 336–362. 10.1086/227461

[B117] Kemper T. D. (1986). How many emotions are there? Wedding the social and autonomic competences. Am. J. Sociol. 94, 263–289.

[B118] Kemper T. D. (1991). Predicting emotions from social relations. Soc. Psychol. Q. 54, 330–342. 10.2307/2786845

[B119] Kilner J. M. Neal A. Weiskopf N. Friston K. J. Frith C. D. (2009). Evidence of mirror neurons in human inferior frontal gyrus. J. Neurosci. 29, 10153–10159. 10.1523/JNEUROSCI.2668-09.2009 19675249 PMC2788150

[B120] Knutson K. M. Mah L. Manly C. F. Grafman J. (2007). Neural correlates of automatic beliefs about gender and race. Hum. Brain Mapp. 28, 915–930. 10.1002/hbm.20320 17133388 PMC6871386

[B121] Kohler E. Keysers C. Umilta M. A. Fogassi L. Gallese V. Rizzolatti G. (2002). Hearing sounds, understanding actions: action representation in mirror neurons. Science 297, 846–848. 10.1126/science.1070311 12161656

[B122] Krienen F. M. Tu P. C. Buckner R. L. (2010). Clan mentality: evidence that the medial prefrontal cortex responds to close others. J. Neurosci. 30, 906–915. 10.1523/JNEUROSCI.2180-10.2010 20943931 PMC2989424

[B123] Kringelbach M. L. Berridge K. C. (2009). Towards a functional neuroanatomy of pleasure and happiness. Trends Cogn. Sci. 13, 479–487. 10.1016/j.tics.2009.08.006 19782634 PMC2767390

[B124] Kubota J. T. Banaji M. R. Phelps E. A. (2012). The neuroscience of race. Nat. Neurosci. 15, 940–948. 10.1038/nn.3136 22735516 PMC3864590

[B125] Kurata K. (1993). Premotor cortex of monkeys: set-and movement-related activity reflecting amplitude and direction of wrist movements. J. Neurophysiol. 69, 187–200. 10.1152/jn.1993.69.1.187 8433130

[B126] Kurzban R. Tooby J. Cosmides L. (2001). Can race be erased? Coalitional computation and social categorization. Proc. Natl. Acad. Sci. U.S.A. 98, 15387–15392. 10.1073/pnas.251541498 11742078 PMC65039

[B127] Lamont M. (1992). Money, Morals, and Manners: The Culture of the French and the American Upper-Middle Class. Chicago: University of Chicago Press. 10.7208/chicago/9780226922591.001.0001

[B128] Lamont M. (2000). The Dignity of Working Men: Morality and the Boundaries of Race. Class and Immigration. Cambridge, MA: Harvard University Press.

[B129] Lawler E. J. (2001). An affect theory of social exchange. Am. J. Sociol. 107, 321–352. 10.1086/324071

[B130] Lawler E. J. Thye S. R. Yoon J. (2000). Emotion and group cohesion in productive exchange. Am. J. Sociol. 106, 616–657. 10.1086/318965

[B131] Lieberman M. Hariri A. Jarcho J. M. Eisenberger N. I. Bookheimer S. Y. (2005). An fMRI investigation of race related amygdala activity in African-American and Caucasian-American individuals. Nat. Neurosci. 8, 720–723. 10.1038/nn1465 15880106

[B132] Lieberman M. D. (2007). Social cognitive neuroscience: a review of core processes. Annu. Rev. Psychol. 58, 259–289. 10.1146/annurev.psych.58.110405.085654 17002553

[B133] MacDonald A. W. Cohen J. D. Stenger V. A. Carter C. S. (2000). Dissociating the role of the dorsolateral prefrontal and anterior cingulate cortex in cognitive control. Science 288, 1835–1838. 10.1126/science.288.5472.1835 10846167

[B134] Macmillan M. (2000). Restoring phineas gage: a 150th retrospective. J. Hist. Neurosci. 9, 46–66. 10.1076/0964-704X(200004)9:1;1-2;FT046 11232349

[B135] Maess B. Koelsch S. Gunter T. C. Friederici A. D. (2001). Musical syntax is processed in Broca's area: an MEG study. Nat. Neurosci. 4, 540–545. 10.1038/87502 11319564

[B136] Majka P. Kublik E. Furga G. Wójcik D. K. (2012). Common atlas format and 3D brain atlas reconstructor: infrastructure for constructing 3D brain atlases. Neuroinformatics 10, 181–197. 10.1007/s12021-011-9138-6 22227717 PMC3325030

[B137] Maloney P. (2013). Online networks and emotional energy: How pro-anorexic websites use interaction ritual chains to (re) form identity. Inform. Commun. Soc. 16, 105–124. 10.1080/1369118X.2012.659197

[B138] Martin E. (2004). Talking back to neuro-reductionism, in Cultural Bodies: Ethnography and Theory, eds Thomas H. Ahmed J. (Oxford: Blackwell Publishing), 190–211. 10.1002/9780470775837.ch8

[B139] Mead G. H. (1934). Mind, Self and Society From the Standpoint of a Social Behaviorist. Chicago: University of Chicago Press.

[B140] Melamed D. Kalkhoff W. Han S. Li X. (2017). The neural bases of status-based influence. Socius 3:2378023117709695. 10.1177/2378023117709695

[B141] Miller E. K. Cohen J. D. (2001). An integrative theory of prefrontal cortex function. Annu. Rev. Neurosci. 24, 167–202. 10.1146/annurev.neuro.24.1.167 11283309

[B142] Milligan K. Astington J. W. Dack L. A. (2007). Language and theory of mind: Meta-analysis of the relation between language ability and false-belief understanding. Child Dev. 78, 622–646. 10.1111/j.1467-8624.2007.01018.x 17381794

[B143] Milner B. (1963). Effects of different brain lesions on card sorting: the role of the frontal lobes. Arch. Neurol. 9, 90–100. 10.1001/archneur.1963.00460070100010

[B144] Moll J. Eslinger P. J. Oliveira-Souza R. D. (2001). Frontopolar and anterior temporal cortex activation in a moral judgment task: preliminary functional MRI results in normal subjects. Arq. Neuropsiquiatr. 59, 657–664. 10.1590/S0004-282X2001000500001 11593260

[B145] Mushiake H. Inase M. Tanji J. (1991). Neuronal activity in the primate premotor, supplementary, and precentral motor cortex during visually guided and internally determined sequential movements. J. Neurophysiol. 66, 705–718. 10.1152/jn.1991.66.3.705 1753282

[B146] Musso M. Moro A. Glauche V. Rijntjes M. Reichenbach J. Büchel C.. (2003). Broca's area and the language instinct. Nat. Neurosci. 6, 774–7841. 10.1038/nn1077 12819784

[B147] O'Doherty J. Winston J. Critchley H. Perrett D. Burt D. M. Dolan R. J. (2003). Beauty in a smile: the role of medial orbitofrontal cortex in facial attractiveness. Neuropsychologia 41, 147–155. 10.1016/s0028-3932(02)00145-8 12459213

[B148] Omi M. Winant H. A. (1994). Racial Formation in the United States: From the 1960s to the 1990s. New York, NY: Routledge.

[B149] Paus T. (1996). Location and function of the human frontal eye-field: a selective review. Neuropsychologia 34, 475–483. 10.1016/0028-3932(95)00134-4 8736560

[B150] Petrides M. Cadoret G. Mackey S. (2005). Orofacial somatomotor responses in the macaque monkey homologue of Broca's area. Nature 435, 1235–1238. 10.1038/nature03628 15988526

[B151] Phelps E. A. O'Connor K. J. Cunningham W. A. Funayama E. S. Gatenby J. C. Gore J. C.. (2000). Performance on indirect measures of race evaluation predicts amygdala activation. J. Cogn. Neurosci. 12, 729–738. 10.1162/089892900562552 11054916

[B152] Pickersgill M. (2013). The social life of the brain: neuroscience in society. Curr. Soc. 61, 322–340. 10.1177/0011392113476464 24285875 PMC3835147

[B153] Porter R. Lemon R. (1993). Corticospinal Function and Voluntary Movement. Oxford: Oxford University Press.

[B154] Preston S. D. De Waal F. B. (2002). Empathy: its ultimate and proximate bases. Behav. Brain Sci. 25, 1–20. 10.1017/s0140525x02000018 12625087

[B155] Price J. L. (1999). Prefrontal cortical networks related to visceral function and mood. Ann. N. Y. Acad. Sci. 877, 383–396. 10.1111/j.1749-6632.1999.tb09278.x 10415660

[B156] Pyers J. E. Senghas A. (2009). Language promotes false-belief understanding: evidence from learners of a new sign language. Psychol. Sci. 20, 805–812. 10.1111/j.1467-9280.2009.02377.x 19515119 PMC2884962

[B157] Rao S. M. Bandettini P. A. Binder J. R. Bobholz J. A. Hammeke T. A. Stein E. A.. (1996). Relationship between finger movement rate and functional magnetic resonance signal change in human primary motor cortex. J. Cereb. Blood Flow Metabol. 16, 1250–1254. 10.1097/00004647-199611000-00020 8898698

[B158] Raz A. (2004). Anatomy of attentional networks. Anat. Rec. 281B, 21–36. 10.1002/ar.b.20035 15558781

[B159] Richeson J. A. Baird A. A. Gordon H. L. Heatherton T. F. Wyland C. L. Trawalter S.. (2003). An fMRI investigation of the impact of interracial contact on executive function. Nat. Neurosci. 6, 1323–1328. 10.1038/nn1156 14625557

[B160] Rizzolatti G. Arbib M. A. (1998). Language within our grasp. Trends Neurosci. 21, 188–194. 10.1016/S0166-2236(98)01260-0 9610880

[B161] Rizzolatti G. Craighero L. (2004). The mirror-neuron system. Annu. Rev. Neurosci. 27, 169–192. 10.1146/annurev.neuro.27.070203.144230 15217330

[B162] Rizzolatti G. Fadiga L. Gallese V. Fogassi L. (1996). Premotor cortex and the recognition of motor actions. Cogn. Brain Res. 3, 131–141. 10.1016/0926-6410(95)00038-0 8713554

[B163] Robinson D. T. Smith-Lovin L. (2006). Affect Control Theory, in Contemporary Social Psychological Theories, ed P. Burke (Stanford, CA: Stanford University Press), 137–164.

[B164] Roland P. E. Larsen B. Lassen N. A. Skinhoj E. (1980). Supplementary motor area and other cortical areas in organization of voluntary movements in man. J. Neurophysiol. 43, 118–136. 10.1152/jn.1980.43.1.118 7351547

[B165] Rolls E. T. Kringelbach M. L. De Araujo I. E. (2003). Different representations of pleasant and unpleasant odours in the human brain. Eur. J. Neurosci. 18, 695–703. 10.1046/j.1460-9568.2003.02779.x 12911766

[B166] Ronquillo J. Denson T. F. Lickel B. Lu Z. L. Nandy A. Maddox K. B. (2007). The effects of skin tone on race-related amygdala activity: an fMRI investigation. Soc. Cogn. Affect. Neurosci. 2, 39–44. 10.1093/scan/nsl043 18985117 PMC2555431

[B167] Rothbart M. Taylor M. (1992). Category labels and social reality: Do we view social categories as natural kinds?. in Language, Interaction and Social Cognition, eds Semin G. R. Fiedler K. (Thousand Oaks, CA: Sage Publications), 11–36.

[B168] Sanes J. N. (2003). Neocortical mechanisms in motor learning. Curr. Opin. Neurobiol. 13, 225–231. 10.1016/S0959-4388(03)00046-1 12744978

[B169] Sanes J. N. Donoghue J. P. (2000). Plasticity and primary motor cortex. Annu. Rev. Neurosci. 23, 393–415. 10.1146/annurev.neuro.23.1.393 10845069

[B170] Saver J. L. Damasio A. R. (1991). Preserved access and processing of social knowledge in a patient with acquired sociopathy due to ventromedial frontal damage. Neuropsychologia 29, 1241–1249. 10.1016/0028-3932(91)90037-9 1791934

[B171] Saxe R. Carey S. Kanwisher N. (2004). Understanding other minds: linking developmental psychology and functional neuroimaging. Annu. Rev. Psychol. 55, 87–124. 10.1146/annurev.psych.55.090902.142044 14744211

[B172] Schall J. D. (2002). The neural selection and control of saccades by the frontal eye field. Philos. Trans. R Soc. Lond. B. 357, 1073–1082. 10.1098/rstb.2002.1098 12217175 PMC1693021

[B173] Schurz M. Radua J. Aichhorn M. Richlan F. Perner J. (2014). Fractionating theory of mind: a meta-analysis of functional brain imaging studies. Neurosci. Biobehav. Rev. 42, 9–34. 10.1016/j.neubiorev.2014.01.009 24486722

[B174] Scott-Phillips T. C. Dickins T. E. West S. A. (2011). Evolutionary theory and the ultimate–proximate distinction in the human behavioral sciences. Persp. Psychol. Sci. 6, 38–47. 10.1177/1745691610393528 26162114

[B175] Shamay-Tsoory S. G. Harari H. Aharon-Peretz J. Levkovitz Y. (2010). The role of the orbitofrontal cortex in affective theory of mind deficits in criminal offenders with psychopathic tendencies. Cortex 46, 668–677. 10.1016/j.cortex.2009.04.008 19501818

[B176] Singer T. (2006). The neuronal basis and ontogeny of empathy and mind reading: review of literature and implications for future research. Neurosci. Biobehav. Rev. 30, 855–863. 10.1016/j.neubiorev.2006.06.011 16904182

[B177] Smith-Lovin L. (1990). Emotion as the confirmation and disconfirmation of identity: an affect control model, in Research Agendas in the Sociology of Emotions, ed T Kemper (Albany, NY: State University of New York Press), 238–270.

[B178] Smyrnis N. Taira M. Ashe J. Georgopoulos A. P. (1992). Motor cortical activity in a memorized delay task. Exp. Brain Res. 92, 139–151. 10.1007/BF00230390 1486948

[B179] Stanley D. Phelps E. Banaji M. (2008). The neural basis of implicit attitudes. Curr. Dir. Psychol. Sci. 17, 164–170. 10.1111/j.1467-8721.2008.00568.x

[B180] Steinbeis N. Haushofer J. Fehr E. Singer T. (2014). Development of behavioral control and associated vmPFC–DLPFC connectivity explains children's increased resistance to temptation in intertemporal choice. Cereb. Cort. 26, 32–42. 10.1093/cercor/bhu167 25100855

[B181] Stuss D. T. Benson D. F. (1984). Neuropsychological studies of the frontal lobes. Psychol. Bull. 95:3. 10.1037/0033-2909.95.1.3 6544432

[B182] Tafjel H. Turner J. C. (1986). The social identity theory of intergroup behavior, in Psychology of Intergroup Relations, eds Austin W. G. Worchel S. (Chicago, IL: Nelson-Hall), 7–24.

[B183] Tanji J. (1994). The supplementary motor area in the cerebral cortex. Neurosci. Res. 19, 251–268. 10.1016/0168-0102(94)90038-8 8058203

[B184] Tanji J. Shima K. (1994). Role for supplementary motor area cells in planning several movements ahead. Nature 371, 413–416. 10.1038/371413a0 8090219

[B185] TenHouten W. D. (1973). Science and its Mirror Image: A Theory of Inquiry. New York, NY: Harper and Row.

[B186] Thoits P. A. (1989). The sociology of emotions. Annu. Rev. Sociol. 15, 317–342. 10.1146/annurev.so.15.080189.001533

[B187] Turner J. H. Maryanski A. (2012). The biology and neurology of group processes, in Biosociology and Neurosociology, eds Kalkhoff W. Thye S. R. Lawler E. J. (Bingley: Emerald Group Publishing Limited), 1–37. 10.1108/S0882-6145(2012)0000029004

[B188] Uddin L. Q. Iacoboni M. Lange C. Keenan J. P. (2007). The self and social cognition: the role of cortical midline structures and mirror neurons. Trends Cogn. Sci. 11, 153–157. 10.1016/j.tics.2007.01.001 17300981

[B189] Vaidyanathan B. Strand M. Choi-Fitzpatrick A. Buschman T. Davis M. Varela A. (2016). Causality in contemporary American sociology: an empirical assessment and critique. J. Theory Soc. Behav. 46, 3–26. 10.1111/jtsb.12081

[B190] Volz K. G. Kessler T. von Cramon D. Y. (2009). Ingroup as part of the self: Ingroup favouritism is mediated by medial prefrontal cortex activation. Soc. Neurosci. 4, 244–260. 10.1080/17470910802553565 19085561

[B191] Waxman S. G. (2010). Clinical Neuroanatomy, 26th Edn. New York, NY: McGraw-Hill Medical.

[B192] Weinrich M. Wise S. P. (1982). The premotor cortex of the monkey. J. Neurosci. 2, 1329–1345. 10.1523/JNEUROSCI.02-09-01329.1982 7119878 PMC6564318

[B193] Wellman Jr J. K. Corcoran K. E. Stockly-Meyerdirk K. (2014). God is like a drug…: Explaining interaction ritual chains in American megachurches. Soc. Forum. 29, 650–672. 10.1111/socf.12108

[B194] Wellman H. M. Cross D. Watson J. (2001). Meta-analysis of theory-of-mind development: the truth about false belief. Child Dev. 72, 655–684. 10.1111/1467-8624.00304 11405571

[B195] Wexler B. E. (2006). Brain and Culture: Neurobiology, Ideology, and Social Change. Cambridge, MA: MIT Press. 10.7551/mitpress/1658.001.0001

[B196] Wills J. FeldmanHall O. NYUPROSPEC Collaboration Meager, M. R. Van Bavel J. J. (2018). Dissociable contributions of the prefrontal cortex in group-based cooperation. Soc. Cogn. Affect. Neurosci. 13, 349–356. 10.1093/scan/nsy023 29618117 PMC5928404

[B197] Wimmer H. Perner J. (1983). Beliefs about beliefs: Representation and constraining function of wrong beliefs in young children's understanding of deception. Cognition 13, 103–128. 10.1016/0010-0277(83)90004-5 6681741

[B198] Wise S. P. (1985). The primate promoter cortex fifty years after Fulton. Behav. Brain Res. 18, 79–88. 10.1016/0166-4328(85)90064-6 3938285

[B199] Young L. Camprodon J. A. Hauser M. Pascual-Leone A. Saxe R. (2010). Disruption of the right temporoparietal junction with transcranial magnetic stimulation reduces the role of beliefs in moral judgments. Proc. Natl. Acad. Sci. U.S.A. 107, 6753–6758. 10.1073/pnas.0914826107 20351278 PMC2872442

